# Extending the geographic reach of the water hyacinth plant in removal of heavy metals from a temperate Northern Hemisphere river

**DOI:** 10.1038/s41598-018-29387-6

**Published:** 2018-07-23

**Authors:** Jonathan L. Jones, Richard O. Jenkins, Parvez I. Haris

**Affiliations:** 1 Faculty of Health & Life Sciences, De Montfort University, The Gateway, Leicester, LE1 9BH United Kingdom; 2 Natural Resources Wales, Maes Newydd, Britannic Way West, Llandarcy, Neath-Port Talbot SA10 6JQ United Kingdom

## Abstract

Water hyacinth *(Eichhornia crassipes)* has been used for environmentally sustainable phytoremediation of water, though its use has been geographically restricted. For the first time we extend its geographical reach by investigating its potential for clean-up of water from a highly polluted British river (Nant-Y-Fendrod, a tributary of the River Tawe). Investigations using the plant were conducted at three levels: a bench-scale study using polluted river water and synthetic solutions; an *in-situ* trial using water hyacinth within the Nant-Y-Fendrod; and a bankside trial to pump and treat river water. The removal of the largest number of heavy metals (21) from water in a single study using ICP-MS is reported, including Sb, for the first time. Results are promising, with bench-scale tests demonstrating up to 63% removal of Al, 62% Zn, 47% Cd, 22% Mn and 23% As, during just seven hours exposure to the plant. When extended to three weeks exposure, removal is evident in the order Al > Cd > Zn > Mn > Ni > As > V. Furthermore, *in-situ* mean removal of 6%, 11% and 15% of Mn, Zn and Cd respectively is demonstrated. As the world learns to adapt to climate change, studies of the type reported here are needed to exploit the remarkable phytoremediation potential of water hyacinth.

## Introduction

*Water hyacinth* is an aquatic plant which originated in the rain forests of the Amazon Basin and is a native plant of Brazil, among other countries in South America. Water hyacinth is the fastest growing free-floating hydrophyte^1^. Records exist of its invasion of the River Nile in the late 18^th^ century and it was subsequently introduced in other countries as a specimen for ornamental ponds and botanical gardens^2,3^.

Water hyacinth has broad, thick, glossy ovate leaves. The leaves are usually 10–20 cm across and float above the water surface on long bulbous stalks. Plants consist of a rosette of six to ten leaves attached to a rhizome with a well-developed, fibrous, root system. The roots are unbranched and have a conspicuous root cap^2^. Water hyacinth also exhibits foliar plasticity, which allows variation in the morphology and physiological function of foliage produced in response to climatic and environmental conditions^3^. It can grow in both saline and freshwater^4^. It reproduces both sexually and by budding and stolen production^5^.

The plant’s attributes create a paradox, in that it is suitable for use as a hyperaccumulator, though its prolific growth rate causes a negative impact upon the environment, human health and economic development in many countries. Rapid growth produces dense mats that can clog water bodies, thus creating blockages that can cause flooding, interfere with navigation, power generation, crop irrigation and ecological status. Conversely, the fact that it is an excellent hyperaccumulator of pollutants, it is abundantly available in many countries, where it is mechanically harvested in attempts to limit its spread and so presents an attractive source as a low-cost, green, remediation strategy. A review by Sharma *et al*. (2016) provides a comprehensive summary of the opportunities and challenges that water hyacinth presents beyond biocontrol^6^.

Phytoremediation of metals using the water hyacinth plant has been ongoing for over four decades (Wolverton 1975)^7^. However, the idea of exploring the phytoremediation potential of this plant within a river beyond its normal habitat has not been previously investigated. This is very important, considering that this plant has proven phytoremediation ability and is also one of the fastest growing plants in the world. In this context, using the plant for phytoremediation in other parts of the world beyond its native habitat needs to be carried out, as it will lay the foundation for future studies where the full potential of plants are utilised by overcoming geographical and climatic limitations. As the world adapts to climate change, it is likely that plants native to one area may flourish in a completely different climatic region. Whilst some studies have investigated growth of a tropical plant in Northern Hemisphere countries, there are no reports to the best of our knowledge, of growing a tropical plant outside of its normal habitat in a Northern Hemisphere river for phytoremediation purposes. To fill a gap in research in this area, we present in this manuscript a pioneering study that investigates the potential of the water hyacinth plant for removal of metals from a highly polluted British river (Nant-Y-Fendrod) at three different levels. Such types of study are also important in that when a plant becomes invasive, a suitable use for it, in place of it being a nuisance requiring costly control mechanisms has several added benefits from a sustainability perspective^8^.

**Figure 1:**
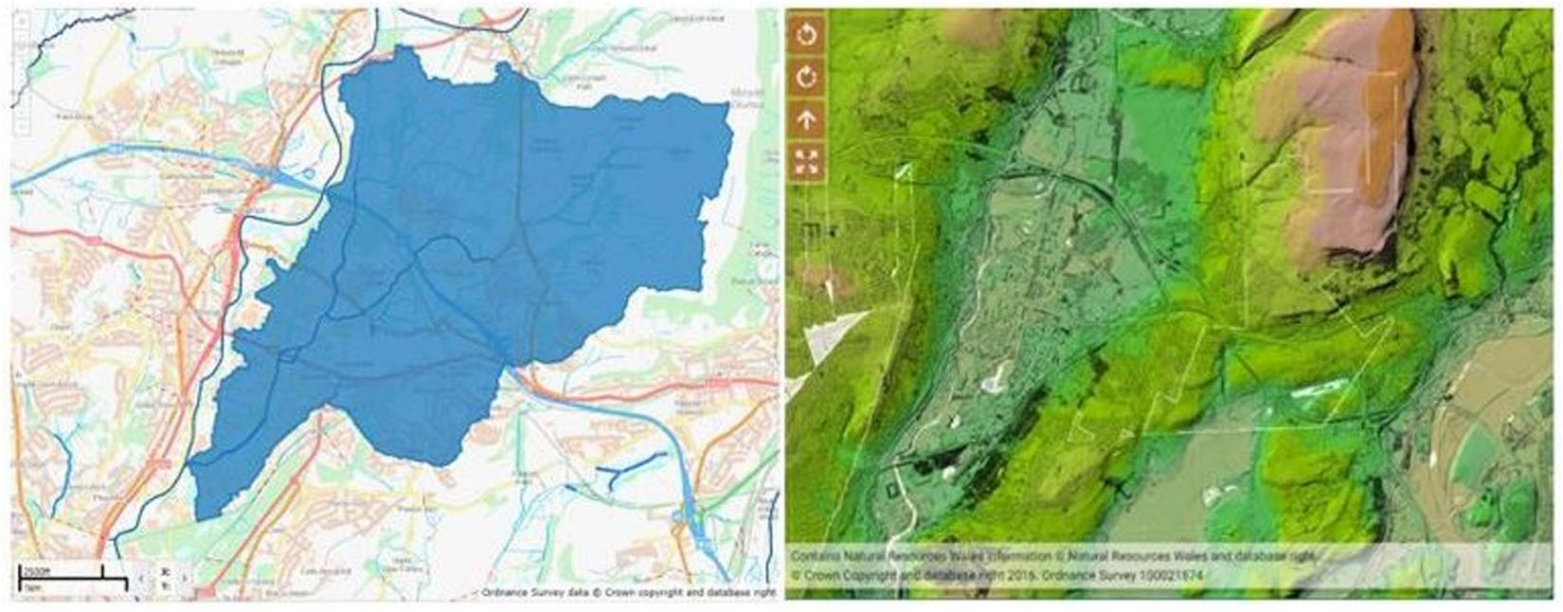
Map showing Nant-Y-Fendrod river catchment area (left) and LIDAR Digital Terrain Model showing topography of the study area (right). Contains Ordinance Survey Data© Crown copyright and database right 2013.

The Nant-Y-Fendrod is a tributary of the River Tawe, which in turn drains the Lower Swansea Valley into Swansea Bay in South Wales (see Fig. 1 for maps). The waterbody flows over land that has a variety of both current and historic land uses, including industrial, agricultural, retail, domestic and recreational use. It has a catchment area of 20 km^2^, is 4.2 km in length and has been heavily modified for the purposes of flood protection and urbanisation. The Fendrod enters the River Tawe on its eastern bank approximately 4.5 km from the coast.

The area is purported to be the birth place of the Industrial Revolution and was a focal point of global copper production during the 19^th^ and 20^th^ Centuries^9^. An estimated 7 million tonnes of copper and zinc smelting waste was abandoned on the valley floor. As land became full of waste, factory owners would acquire more land to dispose of it. Lavender (1981) explained that some smelting techniques were so inefficient that it would be profitable to re-smelt some of the waste arising from the tips^10^. Waste tips caused decimation of vegetation and habitats that this supported. In 1961 the Lower Swansea Valley Project was commissioned, in attempts to remediate the land^11^. Yet, considerable contamination remains, which is affecting water quality.

The standard of ‘Good Ecological Status’ is one of environmental quality imposed by Directive 2000/60/EC^12^. ‘Good Ecological Status’ indicates that a waterbody is generally sustainable and supports healthy plants and wildlife. The classification is based upon the physical, chemical and biological status of a waterbody. Under the WFD, the Fendrod fails to meet standards for ecotoxic zinc, cadmium and manganese.

The current proof of concept study, conducted at three levels, investigated the efficiency of living water hyacinth in the UK climate at the far end of its climatic tolerance for the removal of heavy metal pollutants from Fendrod river water. The study is not only unique in the latter context, but it is also the first study to determine removal of 21 different elements from polluted river water. Water hyacinth is widely regarded as a noxious weed in many parts of the world and indeed, given the currently predicted effects of climate change, governments and regulators of many countries in the Northern Hemisphere have introduced strict legislation to limit and prevent its proliferation. This study extends beyond the level of horizon scanning and preparedness by policy-makers and takes it a step further in examining the exploitation of the plant in turning a problem into a potential solution, before it actually becomes a problem. This radical thinking may influence future research in this field which is timely and necessary.

## Materials and Methods

**Figure 2:**
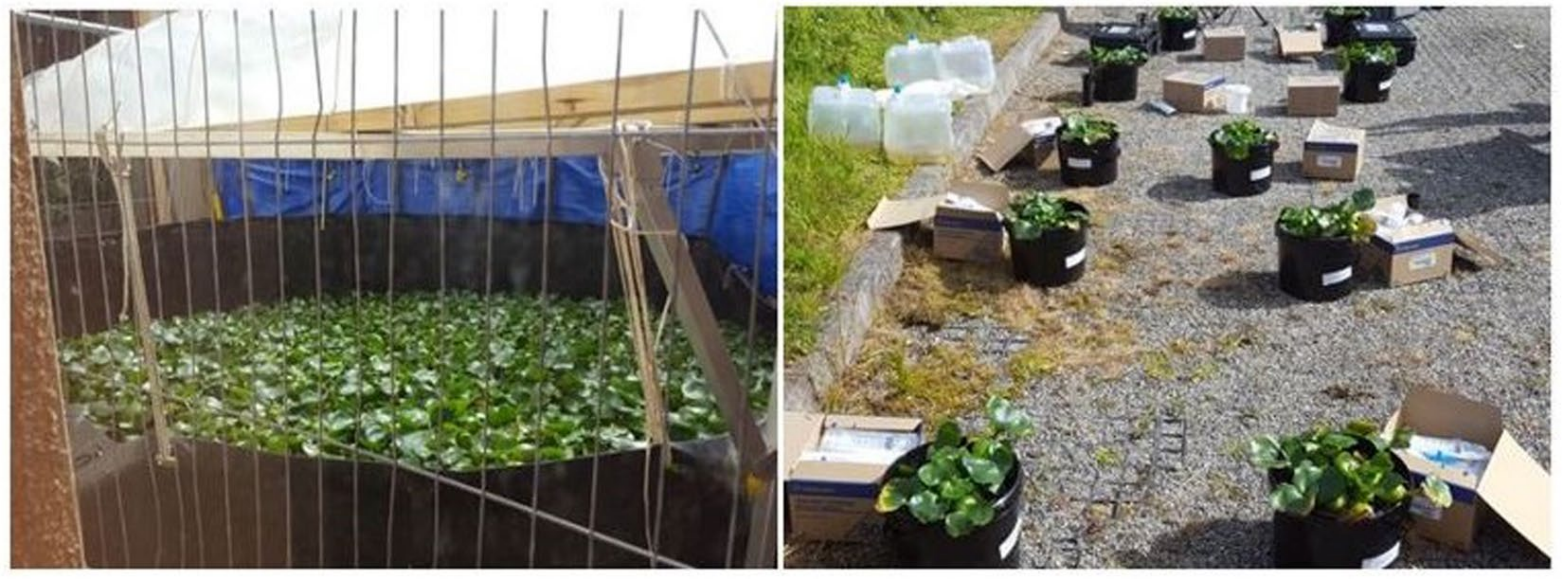
Photographs showing water hyacinth plant containment pond (left image) and Bench-scale study experiment layout (right image).

The water hyacinth plants utilised in all three studies were sourced from a single supplier to ensure good provenance, provide consistency and minimise risk of pre-existing contamination within plant tissue. The supplier, World of Aquatics cultivates the plants at a nursery in Enfield, England UK. Upon receipt, the plants were stored prior to use in a purpose-built containment pond, filled with rainwater and covered with a series of polythene tunnels to retain heat and increase water temperature as can be seen in Fig. 2.

**Figure 3:**
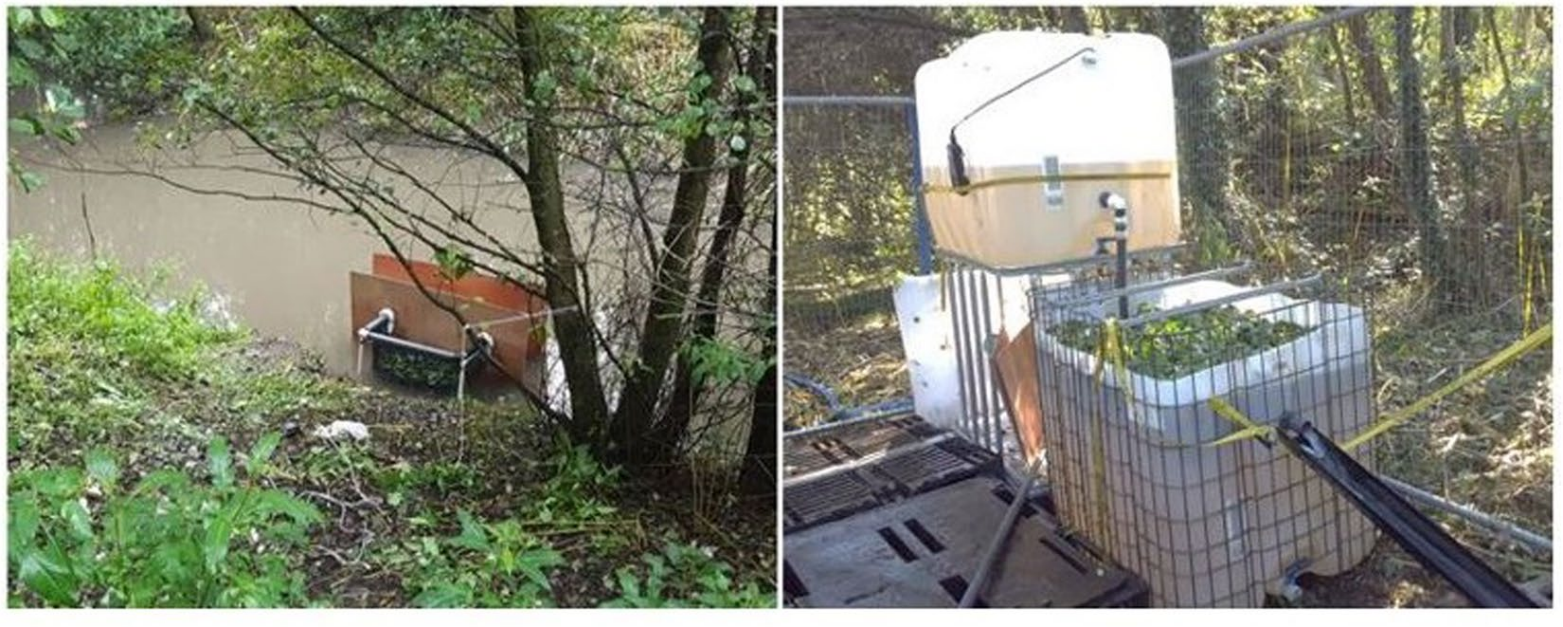
Photographs showing *In-situ* treatment pod within the Nant-Y-Fendrod (left image) and bankside treatment system (right image).

The number of plants used within each of the three studies was broadly based upon the surface area of water to be treated. This is demonstrated within the figures shown of each of the experiments (Figs 2 and 3), where plants effectively cover the surface of the water being treated as they would in their natural setting. The study used only healthy young plants at the bulbous phenostage. Following the plants’ exposure to polluted water, a comprehensive analysis of a total of 21 elements (namely, Be, Al, Ti, V, Cr, Mn, Co, Ni, Cu, Zn, As, Se, Mo, Ag, Cd, Sn, Sb, Te, Tl, Pb and U) was conducted at each level of the study using ICP-MS in order to measure differences observed in upstream/pre-treatment river water and that which had been subjected to treatment by the plants.

### Bench-scale study

An initial study examined the use of water hyacinth in five different ten litre solutions of differing metal concentrations to assess removal efficiency. This was also duplicated with a further five containers to demonstrate repeatability. Figure 2 shows a photograph of the experiment layout. Individual, 11 litre sterile (food grade) plastic containers held the following samples: Solution A, a synthetic solution representing the highest observed levels of zinc contamination derived from water quality monitoring within the Fendrod Stream; Solution B, a synthetic solution representing the average observed levels of zinc contamination; The Nant-Y-Fendrod downstream of the Nant-Y-Ffin (actual river water from a site representative of the highest metal concentrations observed, referred to as site 1 in results); The Nant-Y-Fendrod downstream of the flood alleviation lake (actual river water from a site representative of average metal concentrations observed – site 2); and a control sample containing only mains supply drinking water (with only trace amounts of other substances present). Details of the pollution in the Fendrod and associated water quality assessment can be found in Jones *et al*.^13^.

### General procedure for preparation of zinc solution

Two synthetic solutions, Solution A and Solution B, were prepared to represent the average and maximum zinc-contaminated stretches of the Fendrod Stream respectively. A mass of zinc acetate di-hydrate was calculated using mean values of data collected during six month’s water quality sampling on the Fendrod stream itself. 5 litres of deionised water were spiked with 13.7 mL of the stock solution to make up Solution A and 2.02 mL of the solution was added to 5 litres of deionised water to make up solution B. This yielded a concentration of 4.05 mg/L (99.5% accuracy) for Solution A. Solution B yielded a concentration of 0.586 mg/L (98.3% accuracy). Both in excellent agreement with the calculated values required.

Two individual water hyacinth adult plants, at the “bulbous” phenostage (young, green and healthy) were introduced to each of the containers. Water samples were obtained from each of the containers prior to introduction, then hourly thereafter for a period of seven hours. Samples for dissolved metal analysis were passed through single-use encapsulated filters with a 0.45 µm pore space using sterile syringes. Water temperature, pH, electrical conductivity, dissolved oxygen and salinity were concurrently recorded using a YSI professional plus multi-parameter meter. Samples were also obtained at the start and end of the experiment to measure parameters such as BOD, COD and ammoniacal nitrogen (NH_3_-N).

Samples were analysed at the Natural Resources Wales National Laboratory, accredited to ISO/IEC 17025:2005 by the United Kingdom Accreditation Service. All methods used, including those for analysis of BOD, COD, ammonia and total organic nitrogen (TON) are in strict accordance with the relevant British Standards and Standing Committee of Analysts Blue Book of methods for the examination of Waters and Associated materials^14^.

Metals were analysed using Inductively Coupled Plasma-Mass Spectrometry (ICP-MS). The instrument used was a X Series 2 manufactured by Thermo Fisher. A combined Total method was used, which includes the following metals: Be, Al, Ti, V, Cr, Mn, Co, Ni, Cu, Zn, As, Se, Mo, Ag, Cd, Sn, Sb, Te, Ba, Pb and U. Ga, Rh and Ir were used as internal standards.

The experiment was conducted on three separate occasions using different, previously unexposed, water hyacinth plants from the same batch. The experiment ran for eight hours on the first two days, with the third experiment being prolonged for a period of 21 days in place of just eight hours. The containers were stored securely outside at ambient temperatures, though for the latter, under cover (an open-fronted and open-bottomed clear Perspex shelter) to prevent rainwater ingress, whilst allowing UV penetration. The third prolonged test was in attempt to observe optimal removal rates as cited in some of the previous similar work done in other countries, where optimal removal was reported at approximately 21 days. The three experiments provided a total of 480 observations.

Percentage removal of metals was calculated by using the following equation:

1 $$\begin{array}{c}\frac{(Initial\,Concentration-Effluent\,Concentration)}{Initial\,Concentration}\times 100\\ \,\,\,=\,{\rm{Percentage}}\,{\rm{Removal}}\,{\rm{Efficiency}}\end{array}$$

### *In-situ* study

The second study was conducted within the Nant-Y-Fendrod stream itself. The aim of this experiment, to assess metals removal efficiency in a dynamic natural environment with known pollution problems. A purpose-built treatment pod was constructed to house the plants, allowing easy ingress and egress of water, though preventing escape of plants and plant fragments. This was necessary to comply with the Invasive Alien Species Regulations^15^ and requirements of The Wildlife and Countryside Act^16^. An Environmental Permitting (England and Wales) Regulations 2010^17^ Flood Risk Activity Permit was also required from Natural Resources Wales to conduct work within the flood plain of a main river, due to controls on erecting any structure (whether temporary or permanent) in, over or under a main river.

The treatment pod was made using domestic PVC pipework and fittings to construct a frame, which was covered by a narrow-gauge fish net (typically used for lamprey surveying). Plywood partitions were used to provide a form of separation between untreated and treated water and to allow a duplicate experiment to be run concurrently, effectively two separate treatment pods. The pod was placed directly in the watercourse on the bed. Figure 3 shows the pod construction in use within the watercourse.

A total of 50 previously unexposed individual water hyacinth plants, again at the “bulbous” phenostage were used within the treatment pods. 25 in each treatment pod. The plants weighed between 250 and 350 grams each.

Water quality samples were obtained prior to plant introduction and then hourly thereafter from upstream, within the treatment pods and immediately downstream of the treatment pods for a period of seven hours. The experiment was run in duplicate and repeated on three consecutive days. Plants were removed from the watercourse at the end of each day and securely stored in large plastic bags containing a small amount of water from the Nant-Y-Fendrod.

In-field measurements of water temperature, pH, dissolved oxygen, conductivity and salinity were recorded concurrent with each of the hourly samples. Additional measurements including BOD, COD, TSS, DOC, ammonia, chloride and orthophosphate were obtained every other hour during the experiment, giving a total number of 612 individual observations. Removal efficiency was again calculated using equation 1.

### Bank-side study

The third study was conducted at the bankside of the Nant-Y-Fendrod within a temporary purpose-built treatment compound, as illustrated in Fig. 3. This experiment was again aimed at assessing metals removal efficiency within a dynamic environment, using actual river water, though this time transferred via a pump and pipes to pass through containment to prevent the effluent being diluted with surrounding untreated river water, as was expected during the *in-situ* study. This was also to see how such a small-scale treatment system could work and be up-scaled for real-life application.

The treatment system was constructed using two 1000 litre Intermediate Bulk Containers, one elevated above and behind the other using its metal cage, then fitted with PVC pipework to provide gravity transfer of polluted river water from an influent tank and then discharged to soakaway. Water was transferred from the river to the compound using a petroleum-powered water pump and hose work. Fifty plants at the phenostage were introduced to the treatment pod, which were removed at the end of each test and stored at ambient temperatures within an amount of river water in large plastic bags.

This experiment was repeated over three separate days with samples taken half-hourly over four hours. The experiment timing was dictated by logistics, legalities and physical constraints, in that, abstraction of surface water is limited to 20 m^3^ per day without an abstraction licence being required, this along with the volume of equipment being required to be transferred to and from the site and set-up/dismantled each day.

For these same reasons, the experiment was not run in duplicate as with the bench-scale and *in-situ* studies, though all samples were obtained in duplicate giving a total of 372 observations.

Ideally, the experiments would have been conducted earlier in the summer when climatic conditions would have been more favourable for plant productivity. However, the timing of these experiments was severely set-back by the time it took to obtain all necessary permissions – a warning to future researchers looking to do similar experiments. Samples were again obtained from the influent and effluent using the same methods as those employed in the *in-situ* study.

### Data availability statement

Additional supporting data can be made available upon request to the corresponding author.

## Results and Discussion

There is a discernible need to develop sustainable green technology-based remediation approaches for addressing water pollution around the world. However, studies of certain plants with remarkable phytoremediation potential are often restricted to specific geographical regions and attempts to explore their performance in a climate where it normally does not flourish has not been tested, even though such types of studies are important in the context of sustainability and climate change. This restricts the potential advantages offered by certain plants. For example, the water hyacinth plant is considered to be the fastest growing aquatic plant in the world, which is also very effective at removing pollutants from water. However, its use in phytoremediation has been restricted to Southern Hemisphere countries and thereby limiting its full potential. Indeed, it is surprising that despite over four decades of research with this plant, no previous attempts have been made to assess its ability to remove pollutants from water bodies in Northern Hemisphere countries. Here we report for the first time the potential for extending the geographical limit of the water hyacinth for removing pollutants from a British river by carrying out studies at three different levels, namely the bench-scale, *in-situ* and bankside studies. The methodology adopted in the study is also unique as no previous research with the water hyacinth were conducted at three different as presented here.

Our work on water hyacinth in its non-native environment also provides an awareness and basis for further research related to understanding the impact of climate on growth and performance of plants in different latitudes. Should the British climate become conducive to the plant’s possible introduction and survival, our study, prior to a very large increase in temperature, can provide valuable data and information which could be used for comparison with the performance and application of the plant should the climate in the UK change significantly. It is important to deal with the problems that this plant can bring and this should be something the world, especially countries in the northern latitudes, have to think about and conduct further research upon, as research to date in these regions, with this plant, has been scarce. Putting it to beneficial use in solving an additional global problem may provide just the sort of sustainable solution required.

Diverse methods are being developed for water remediation, including use of plant biomass^18–20^. Work by Rezania (2015) and others have reported the potential of water hyacinth in the removal of pollutants from an aqueous solution^21–23^. Das *et al*. (2015) have investigated the physiological response of water hyacinth to cadmium and its phytoremediation potential, whilst Gupta and Balomajunder (2015) have reported its use in chromium removal^24,25^. Singh *et al*. (2015) have utilised a rotary drum composter to remove Zn, Cu, Mn, Fe, Ni, Pb, Cd and Cr from water hyacinth waste used in treating heavily polluted waste waters, which presents an economically viable method for final disposal of the plants following their use in treatment^26^. This form of treatment can covert waste biomass into well stabilised organic matter that could be reused. More encouragingly, Al-Rmalli *et al*. (2005) have demonstrated the potential of using dry water hyacinth biomass for removal of arsenic from water^27^. This finding facilitates the plant’s use in countries with unsuitable climatic conditions, or that may have strict biosecurity legislation that would prevent introduction into the wild. Again, this would present a cheaper, more environmentally friendly alternative to chemical and mechanical processes of water treatment.

Many previous studies have been conducted using single metal pollutants and single aquatic plants^21,22,24,28,29^. The study presented here, is unique in assessing the removal of several elements from polluted river water with multiple metals and other nutrients present, under both bench-scale and *in-situ* conditions. It is also unique in the sense that water hyacinth, which is a tropical plant, has never been previously used for remediation of river water in Europe, or in the Northern Hemisphere in general. The results of all three studies are presented below in relation to mean percentage metals removal efficiency of *E. crassipes*.

### Bench-scale Study

Excluding a cloudy, overcast period during the afternoon of the third day, weather conditions during the bench-scale study were generally good with some of the warmest and sunniest weather of the summer of 2016 experienced.

Over a period of just seven hours, up to 63% removal is demonstrated in aluminium, 62% in zinc, in cadmium 47%, manganese 22%, with some removal of arsenic (23%), nickel (19%) and cobalt (14%) also evident. When extended to three weeks exposure, during the third run of the experiment, 100% removal is evident in cadmium, cobalt and manganese, with 80% removal of zinc. This is in accordance with the findings presented by others such as Hammad (2011), Smolyakov (2012) and Yapoga *et al*. (2013) reported within the review conducted by Rezania *et al*. (2015), which reports optimum removal at around 21 days^21^.

**Figure 4:**
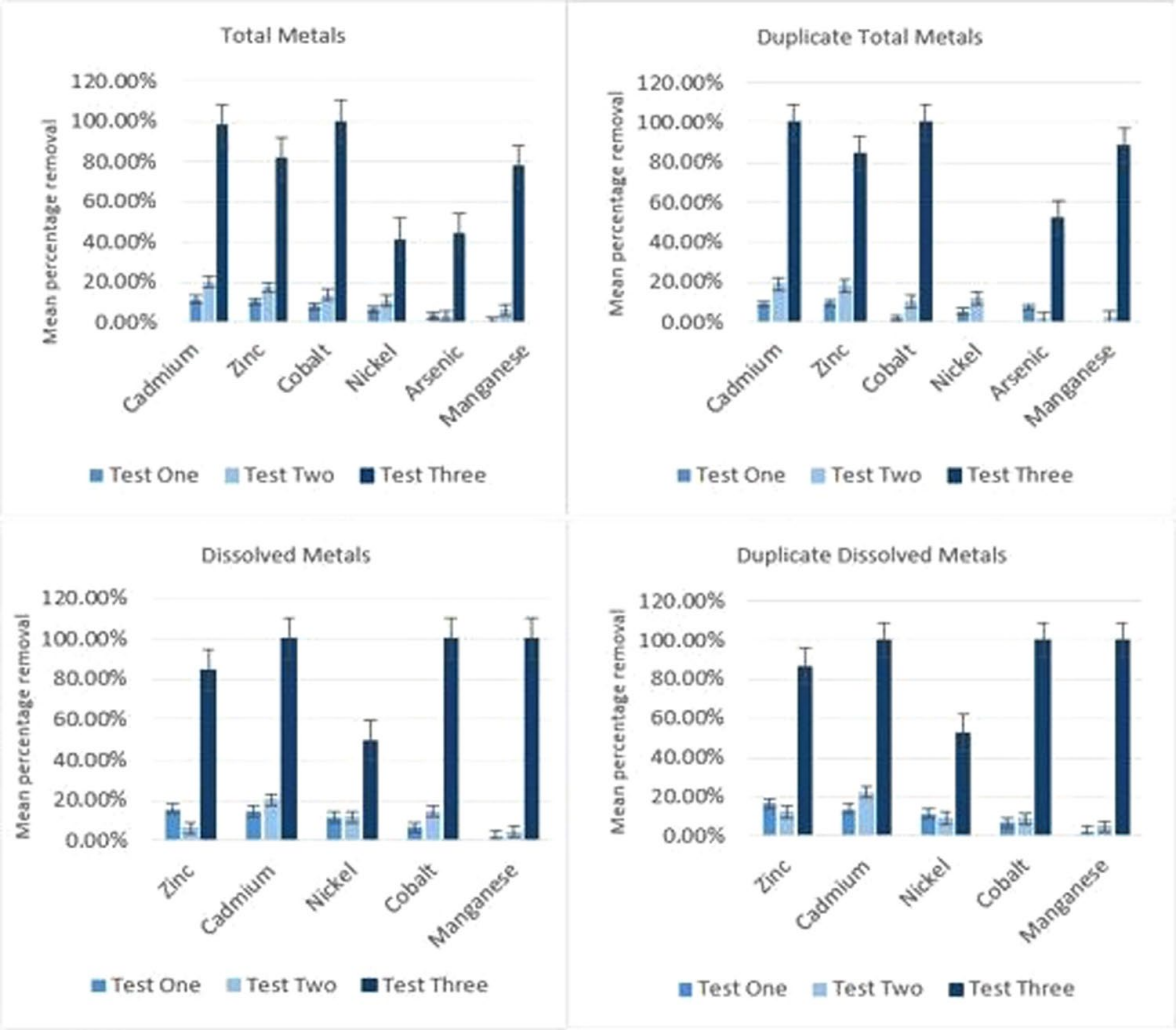
Mean percentage removal of metals by *E. crassipes* in Nant-Y-Fendrod water sample from site 1 (bars denote Standard Error based upon seven replicates). Test 3 results obtained following 21 days exposure. Average Initial metal concentrations in µg/L were as follows: Zn – 2020.33; Ca – 6.23; Ni – 3.99; Co – 1.66; Mn – 421.83; As – 2.11.

The findings of the bench-scale study show that the plants are capable of handling relatively high concentrations of zinc without beginning to show a capacity, or saturation point being reached, with initial zinc concentrations being as high as 4.05 mg/L. The results, which can be seen in Fig. 4 also show the potential for removal of other metals present in the Fendrod at relatively high concentrations, for example, the EQS for cadmium in the Fendrod is a Maximum Allowable Concentration (MAC) EQS of 0.9 µg/L^12^. Initial concentrations of cadmium are several orders of magnitude higher, so such a reduction in cadmium over a short timescale is significant.

**Figure 5:**
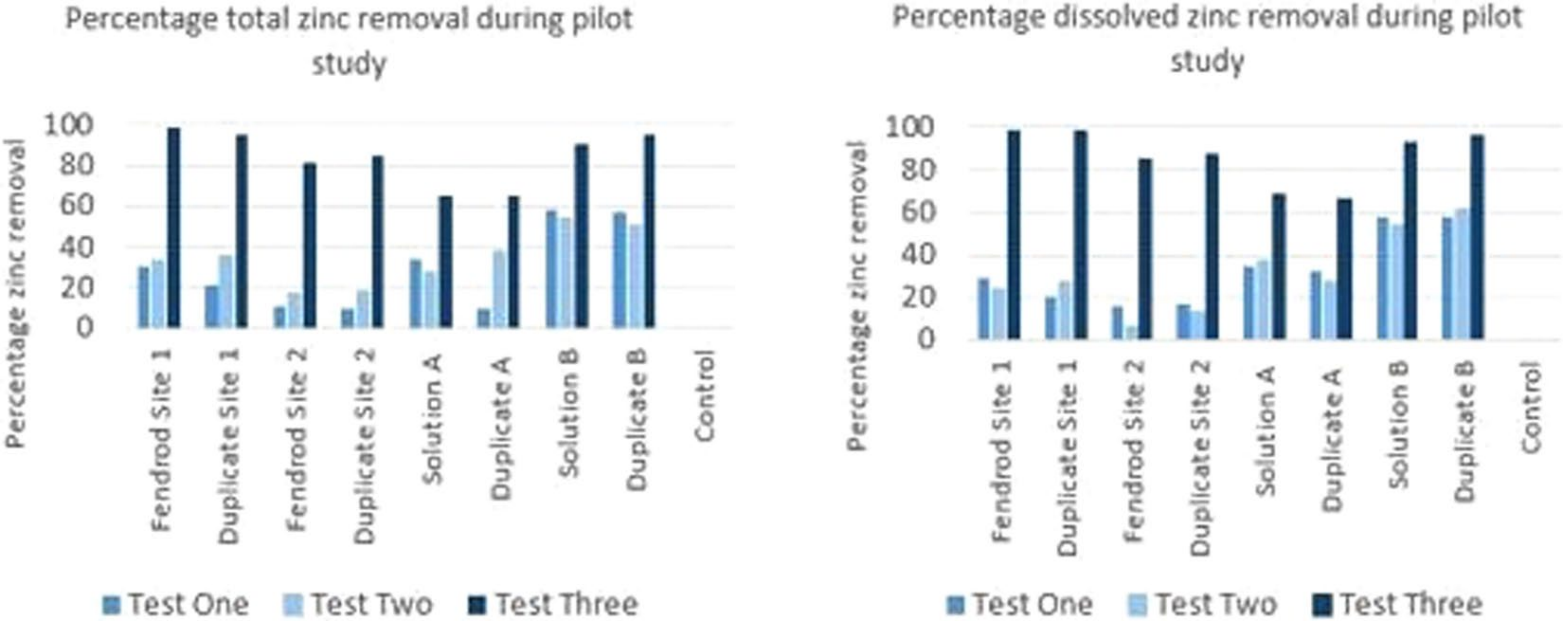
Percentage total and dissolved metal removal by *E. crassipes* during the pilot study. Test 3 results obtained after 21 days exposure. Average initial concentrations in mg/L were as follows: Site 1–2.02; Duplicate Site 1–1.98; Site 2–0.57; Duplicate Site 2–0.51; Solution A – 4.46; Duplicate Solution A – 4.46; Solution B – 0.612; Duplicate Solution B – 0.608; Control – 0.

The results in Fig. 5 show the percentage metal removal results specifically in relation to zinc in the two river water samples and the two synthetic solutions produced for comparison in the absence of other nutrients. The relevant Environmental Quality Standard for zinc in a river with typical hardness of 100–250 mg/L of CaCO_3_ is 0.75 µg/L, so it can be seen from the initial concentrations listed in each of the tables, that levels present in the Fendrod are orders of magnitude higher, often in milligrams per litre. Gaining a significant reduction by hundreds of micrograms per litre over just seven hours demonstrates proof of concept, which provided the impetus for the subsequent *in-situ* study.

### *In-situ* study results

**Figure 6:**
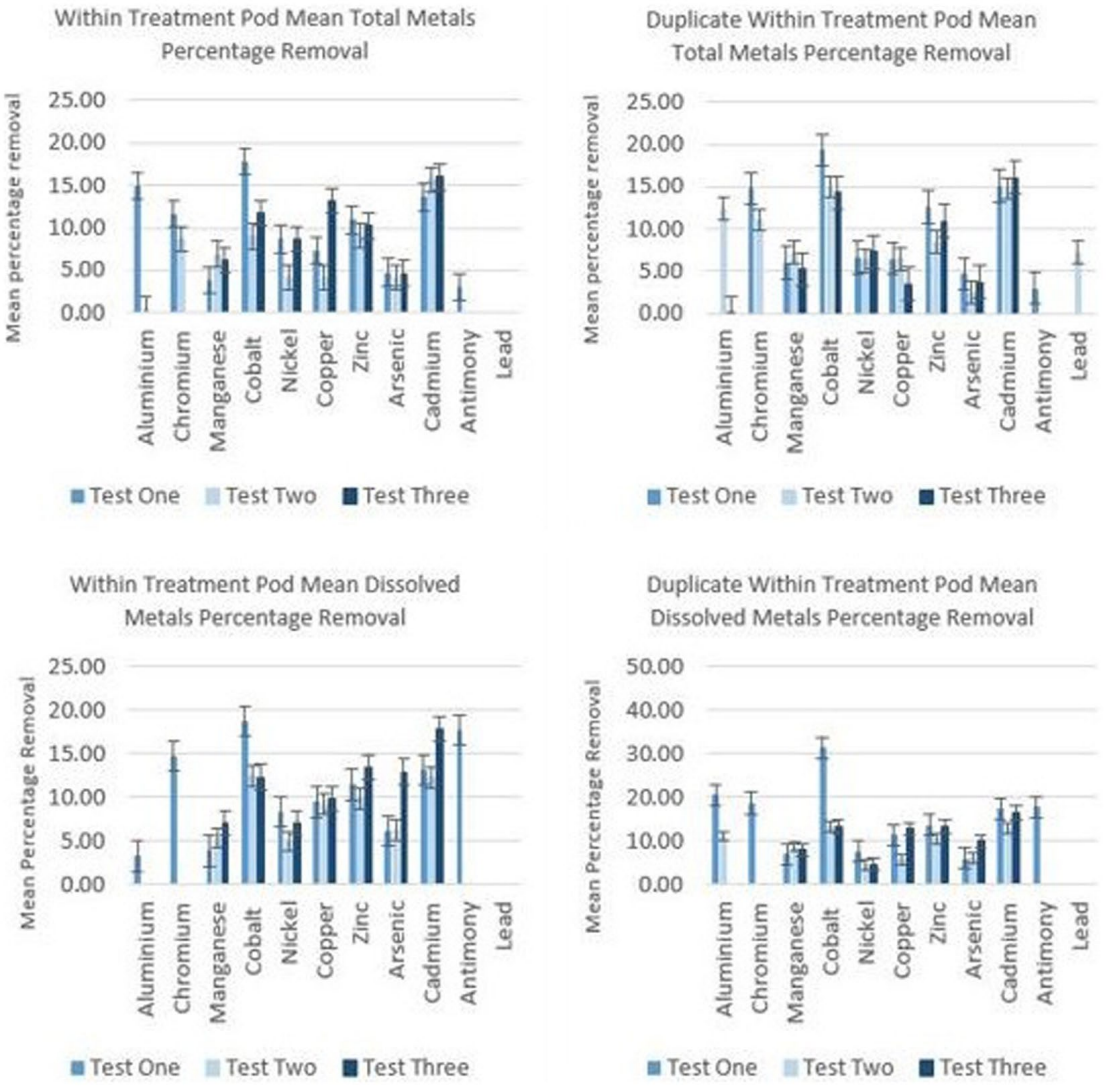
Mean percentage total and dissolved metal removal by *E. crassipes* during *in-situ* study. Bars denote Standard Error based upon seven replicates. Average initial concentrations in µg/L were as follows: Al – 89.82; Cr – 5.56; Mn – 407.3; Co – 2; Nickel – 4.24; Copper – 8.52; Zn – 2032; As – 2.84; Cd – 8.85; Sb – 2.61; Pb – 3.43.

**Figure 7:**
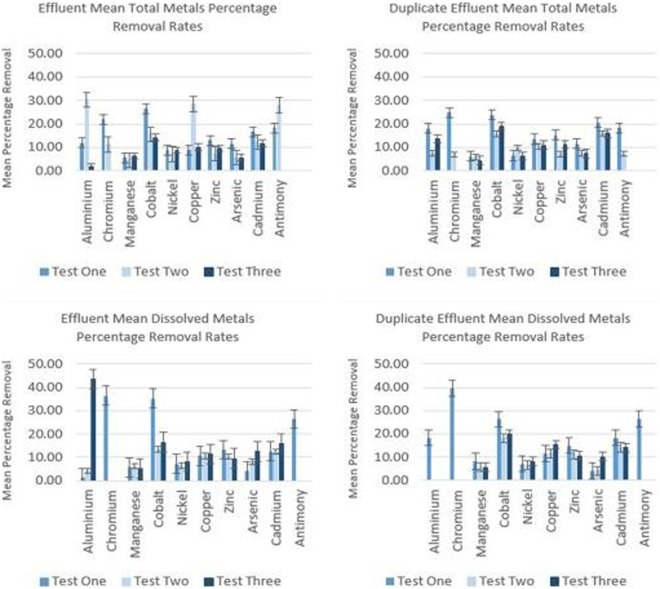
Mean percentage total and dissolved metal removal by E. crassipes within effluent (downstream of treatment pod).

The *in-situ* study was conducted to assess whether the plants can remove metal pollutants from actual polluted river water that is flowing in a dynamic environmental setting. The results presented in Figs 6 and 7 are encouraging, in that an amount of removal is demonstrated, given only a very short exposure time. Also, is the fact that this experiment was not conducted during the peak of summer, when the growth of the plant will be at its maximum. It is possible that maximum removal will be achieved during UK summer months of June, July and August.

**Figure 8:**
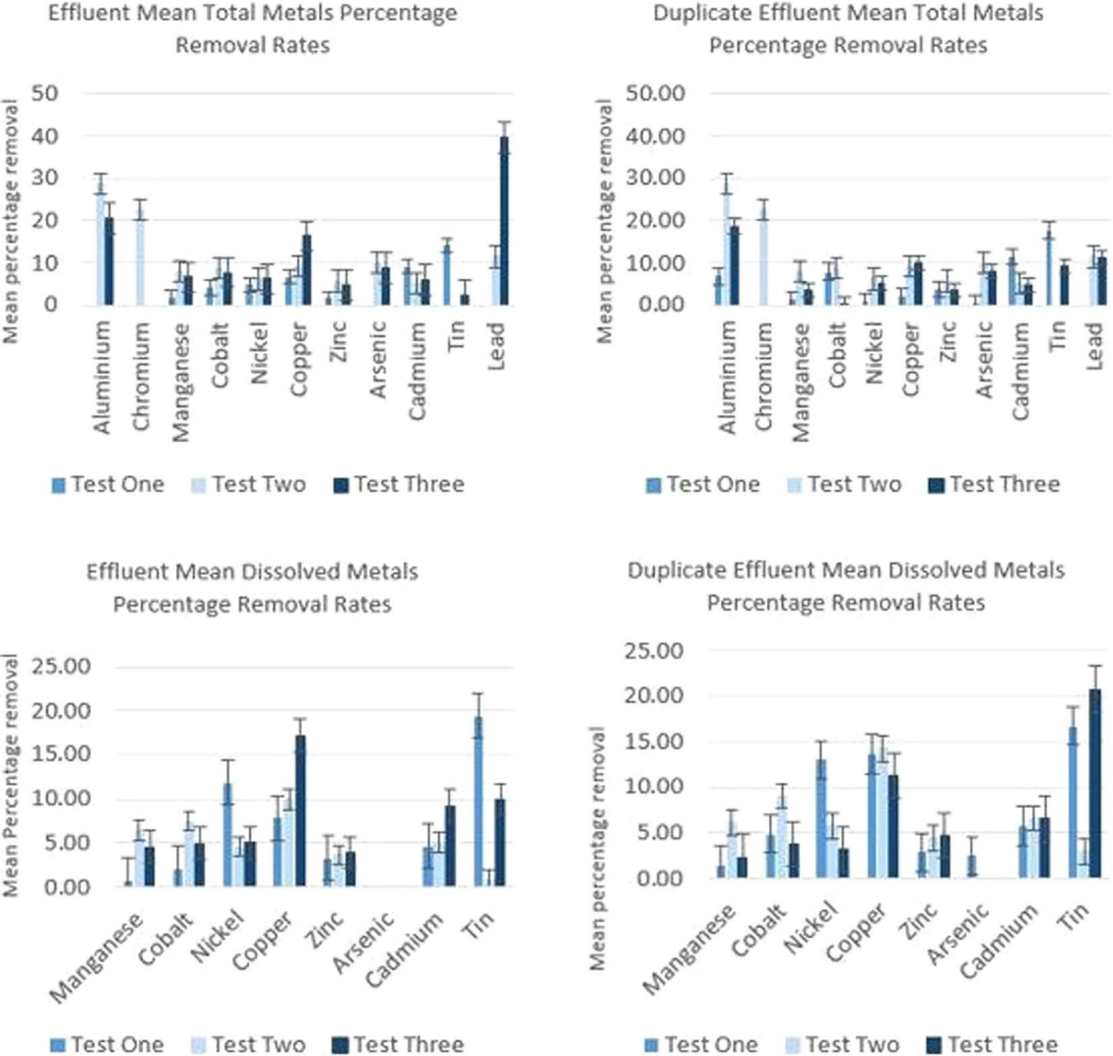
Mean percentage total and dissolved metal removal by *E. crassipes* during the bankside study. Bars represent Standard Error over 8 replicates.

Furthermore, the UK climatic setting is much cooler than that of native countries where the plants may be employed, yet still affords phytoremediation potential. Ideally, plants would be transferred to the river in mid-May and then collected in mid-September, giving a four-month window for phytoremediation. Mean values have been calculated for each of the metals where removal was exhibited. Results were obtained from directly within the treatment pod (shown in Fig. 6), as it was envisaged that mixing of untreated water in direct connectivity with the downstream samples would mask some of the removal taking place by reintroducing metals to the treated water.

Figure 7 shows mean percentage metal removal downstream of the treatment pod. There appears to be only a small amount of interference from untreated water, as these results are not vastly different to those obtained within the treatment pod, even in open connectivity with the untreated water downstream.

Phytoremediation efficiency in plants is generally assessed by calculating the index of their Bioconcentration Factor (BCF). This is calculated by dividing the final metal concentration in the plant tissue by the initial concentrations in water^30^. Plants with a BCF > 1000 are considered good accumulators of heavy metals and this value is used most typically in their selection. *E. crassipes* meets these criteria for some of the metals such as zinc, with the exception of manganese, which exhibits a BCF below 1000. Newete *et al*. (2016), showed that manganese accumulation increased with greater concentrations in the growth medium^31^. The initial concentrations did not vary enough within this study to fully assess this assertion. Within the treatment pod, removal is evident in the order Cr > Cd > Co > Zn > Cu > Al > Ni > Mn > As > Sb and immediately downstream of the treatment pod within the effluent thus: Cr > Co > Sb > Cu > Al > Cd > Zn > Ni > As > Mn. Subtle differences in the order of removal are likely to reflect those of the water chemistry which was dynamic under the in-river conditions. Other factors which may also be an influence are plant biochemistry and the relative concentration, species and oxidation state of metals present at any given time. The initial concentrations of some of the metals present were also far lower than others, in the order of tens, to hundreds and even thousands of micrograms.

Chemical and biological changes occurring within the treatment pod are subtle, yet present in comparison with those of the untreated water. A small increase in pH was observed between the influent and effluent (shown in supplementary data, Fig. S1). pH is the most important parameter in the biosorptive process, in that it affects the solution chemistry of the metals, the activity of the functional groups in the biomass and the competition of metallic ions^28,32–34^. Jayaweera *et al*. (2008) suggest an optimum pH within the growth medium of between 3.5 and 9 for adsorption of metal ions on the roots of water hyacinth^35^. The pH during all three studies was well inside this range at around 7 to 7.8.

Dissolved Oxygen decreases by around 10%. The presence and activity of *E. crassipes* within the treatment pod caused a minor increase in BOD by typically 0.3 mg/L. Prasad *et al*. (2001) demonstrated that mild metal stress has been shown to increase the rate of oxygen consumption through respiration^36^. This is due to an increased energy demand required for ionic transportation at the plasma membranes or the production of specific metal-binding chelates from the sensitive organelles in the cytoplasm (Lösh and Köhl 1999)^29^.

Conductivity decreased as the concentration of ions in the treatment pod was reduced. This is evident in the order of around 40 µS/cm. Total and organic nitrogen and ammonia also decrease slightly as the experiments progressed, which may indicate added benefits in the treatment of organic waste water e.g. sewage effluent. Again, a selection of time series data illustrating some of the changes observed can be found in the supplementary data (Fig. S1–S4).

The level of nutrients present in the form of total organic nitrogen (TON) and phosphorous will also have an impact on the amount of metal uptake. Under nutrient starving conditions (no N or P and other nutrients) within synthetic zinc Solutions A and B, percentage removal efficiency is increased in comparison with the river water samples. This is similar to experiments conducted by Jayaweera *et al*. (2008) who found a progressive decline in the removal of iron with an increase in the level of available nutrients^35^. Jain *et al*. (1990), found that zinc at a concentration of between 5 and 10 mg/L had a favourable effect on the growth of water hyacinth, which may be due to the plants utilising Zn as micronutrient^37^.

### Bankside study

The bankside study was subsequently designed as a small-scale treatment plant that would treat polluted water under continuous flow conditions, as would be required to treat river pollution. At the same time, accurate measurement of metal concentration measurements could be taken without the effluent and treatment pod being “diluted” by surrounding river water, as was the case during the *in-situ* trial. Climatic conditions during this study were not as favourable as those during the pilot and *in-situ* studies, with lower water temperatures between 11 °C and 13 °C and lower ambient temperatures, shorter daylight hours and lower solar UV radiation. This was due to delays in obtaining the relevant permissions to conduct the work, which pushed the test back into the beginning of autumn. Ideally, this test would have been conducted during the warmest longest days of the year. However, it still serves as a useful assessment of this type of phytoremediation in the temperate UK climate and close to the plants’ maximum tolerance. Mean percentage metal removal shown in Figure 8.

The results of this test were again encouraging in that, even under such limiting climatic conditions, almost instantaneous metals removal is evident in the order Pb > Cr > Al > Sn > Cu > Co > Cd > As > Mn > Zn > Ni. The speed and capacity for removal tends to concur with previous studies, where the main mechanism appears to be by adsorption and absorption of metals. In this case, onto the long, hairy root structure of the plants, more so than uptake into the main plant as part of photosynthesis. The finding that the water hyacinth plant is highly effective in the removal of Pb and Al is consistent with previous findings, which reported that the bioconcentration factor of Pb and Al in roots were found to be the highest compared to other detected elements. The bioconcentration of the elements in whole water hyacinth plants reported by Shi & Xia (2010) was recorded in the following order: K (14896.92 µg g-1) > P (5775.78 µg g-1) > S (5596.38 µg g-1) > Ca (2368.92 µg g-1) > *Pb* (2380.03 µg g-1) > *Mn* (852.90 µg g-1) > Fe (782.63 µg g-1) > *Al* (456.26 µg g-1) > *Zn* (134.94 µg g-1) > Sr (84.73 µg g-1) > Mo (26.82 µg g-1) > B (23.16 µg g-1) > *Cr* (10.51 µg g-1) > *Cu* (4.64 µg g-1) > *Ni* (4.49 µg g-1) > *As* (3.94 µg g-1) > *Co* (0.96 µg g-1) > *Cd* (0.23 µg g-1)^38^. The elements we investigated are highlighted and underlined in italic font and as can be seen, there is some similarity in terms of higher uptake of these elements as those reported in our study, which may explain their higher content in the plant. This is highly likely in the case of Pb and Al.

The work by Shi and Xia (2010) also observed that most nutrients and metals present during the study were highly bioconcentrated and that the concentration of the element in the plant was higher when its content in the water was higher^38^.

The amount of metal removal is lower in comparison with the *in-situ* study, though this is likely to be due to the much cooler climatic conditions and potentially shorter contact time between the plant roots and the water being passed through the treatment pod under gravity. Flow rates also varied based upon the water level in the header tank, which had to be intermittently refilled from the river via the water pump.

It is noteworthy that lead is most preferentially removed at times during this study. During the bench-scale and *in-situ* studies, Pb tended to increase throughout the duration of the experiments and did so a lot of the time during this experiment. However, a maximum of 54.7% removal took place at one point during this study, where initial concentrations were 5.48 µg/L reduced to 2.48 µg/L. This merits further investigation, given the deleterious and widespread effects of Pb pollution within the UK and other parts of the world. Like cadmium, lead has no known beneficial metabolic role, though is a known toxin^39^. It is well documented that Pb enters the food chain via biota, particularly fish and via polluted river water used for crop irrigation, which bioaccumulates causing health problems. Both Pb and Cd are regarded as Class B human carcinogens^40^.

Rezania describes work by Liao and Chang (2004)^21^, that ranked water hyacinth metal removal rates of Cu > Zn > Ni > Pb > Cd, which showed highest and lowest removal efficiency being that of copper and cadmium respectively. This is contrary to the results obtained in this study using UK river water, where some of the highest removal rates demonstrated by water hyacinth being that of cadmium. However, work by Yixiong and Xiumin (1990) in a study at Dianchi Lake in China, found that the highest rates of accumulation were for Pb and Cd, which bears greater similarity with our study^41^. Their work also showed that accumulation in the root system is several times higher than that found in the stems or leaves. It is an interesting observation that the order of metal ion removal by the plants varies with each of the different experimental conditions. As with the above described Pb removal, we believe that this is likely to be due to several limiting and controlling factors such as plant provenance and age, climatic conditions, water temperature, site specific water chemistry and differing initial concentrations and oxidation states of species of metals and other nutrients present. With the exception of work by those such as Shi and Xia (2010), Rezania *et al*. (2015) and Newete *et al*. (2016) most previous work has examined removal under nutrient starving conditions, whereas in this study, there are many other nutrients present and available within the river water, as would be the case in a “real” remediation setting within any river^21,31,38^.

The water chemistry of the river and associated samples varied during the differing experiments, being inevitably dynamic and dependent upon many variables including the contemporaneous meteorological conditions such as temperature, precipitation, wind and the associated surface water run-off and groundwater changes that result from these. This in turn, affects the suspended load of particulate matter within the watercourse and the concentrations of any substances that it encounters, or enters into it. This will also influence the pH, hardness and levels of dissolved organic carbon present, which when higher, influences the amount of free ion activity within the water, as reported by Paquin *et al*. (2002) who report that each of these parameters are critical to the bioavailability of dissolved metals in the aquatic environment, which is explainable by their development of the biotic ligand model^42^. In contrast to previous work, it is likely that these subtle changes and associated effects will have influenced the order of metal removal under each of the different conditions tested here.

Subtle differences, such as those within our study are also reported in the various work of others including Newete *et al*. (2016), Shi and Xia (2010), Agunbiade (2008), Harun *et al*. (2008) and Wei *et a*l. (2009)^31,38,43,44^. with similar explanations offered, in that the success of phytoextraction of elements depends on factors such as the degree of site contamination, a plant’s ability to intercept, absorb and accumulate metals in shoots, metal availability for uptake into roots governed by its dissolution into aqueous phase and then interaction between the plant habitat, the metals present and the plant itself, which is complex and controlled by climatic conditions^43,44^. Ernst (1996) reports that the same variability and complexity of the various interactions that take place, make phytoremediation more of a site specific technology than a generic one^45^.

Agunbiade *et al*. (2009) found that the plant accumulated toxic metals such as Cr,Cd, Pb and As both at the root and at the shoot in high degree^34^. They also assessed the translocation of elements, termed translocation factor (TF) in phytoremediation by Baker and Brooks (1989), which is a plant’s ability to transfer pollutants from the root to the plant shoot^46^. This is an important factor in the phytoremediation process and for an effective phytoremediator its TF should be above 1. Their work reported a TF of above 1 for metals such as Zn, Pb, Ni, Mn, Cr, Cu, Cd and As, which is again supported by the removal of those metals within our study.

The fact that hundreds of micrograms can be removed so quickly, could be the difference between a water body passing or failing to meet the relevant Environmental Quality Standards. Due to the limitations in these experiments and initial concentrations in the Fendrod, this is not the case quite yet. However, with further refinement by increasing contact time, for example, by slowing down water flow and affording greater attenuation in perhaps the construction of off-line treatment lagoons, this would be possible. In the case of zinc, Ramesh *et al*. (2003) reported that zinc is borderline acid and therefore can interact strongly with ligands, it is not redox reactive, and it is relatively labile and therefore undergoes ligand exchange reactions rapidly^47^. Tipping and Hurley (1992) in Tipping (1988) describe their humic Ion-Binding Model, which describes the interactions of protons and metal ions with humic substances, which influence their transport and bioavailability^48^. The changes in river water chemistry during this study may have therefore influenced (i) site heterogeneity (discrete sites with a range of affinities, together with the formation of bidendate sites (donate two electrons to a metal atom)) (ii) electrostatic effects (including the contribution of counter-ion accumulation to binding) and (iii) competition among protons and metal ions.

Cadmium is one of the most toxic heavy metals and is considered non-essential for living organisms^49^. Stratford *et al*. (1984) reported that Cd caused substantial reduction in water hyacinth growth by suppressing root development and reducing growth rate by 90% compared to those of controls^50^. Conversely, Dou (1988) found that it may stimulate growth of some plants in small amounts, as may be the case in this study^51^.

The work of Romero-Gonzalez *et al*. (2001) and Brinza *et al*. (2009) (in Agunbiade *et al*. (2009)) reports the translocation of Cd from root to shoot being highest among other metals^34^. The findings of Romero-Gonzalez *et al*. showed that Cd displaces Ca and Na via an ion-exchange mechanism. This may explain its higher removal rate during the bench and *in-situ* studies in contrast with the bankside experiments that took place over a shorter period of time and in cooler UK autumnal conditions.

Zheng *et al*. (2016) in a binary study of Cu and Cd competitive sorption found that Cd was significantly inhibited by Cu^52^. Initial concentrations of Cu in the present study were very low at approximately 8 µg/L. This may account for the higher uptake of cadmium. Ionic exchange has been identified as a predominant mechanism of the metal sorption by water hyacinth roots, so it is likely that the interactive impacts of the various metals present at any time that has led to different competitive sorption during the different experiments.

With specific regard to chromium, Hasan *et al*. (2010) reported that Cr uptake decreased with an increase in pH, though increased with temperature and initial metal concentration^53^. This may explain the differences in Cr removal during the *in-situ* and bankside tests, given the variations in water chemistry over the duration of the experiments.

The findings of this study could prove useful for removal of metal pollutants from water, not only within the UK, though in many other countries where problems of this nature occur. Work by Al-Rmalli *et al*. (2005) has focused on arsenic pollution and heavy metal removal in Bangladesh^27^. Bhuyan and Islam (2017) have further reviewed the effects of heavy metal pollution and its effects upon river and groundwater in Bangladesh^54^. The fact that our study demonstrates Water hyacinth plant can remove Pb, Cr, Cd and As (elements that are known pollutants of Bangladeshi rivers), suggests that this approach could also be applied in Bangladeshi rivers.

Information relating to root vs shoot metal accumulation and adsorption vs absorption in *E. crassipes* is scarce. In general, most studies report higher concentrations of metals in roots than in shoots. Skinner *et al*. (2007) found in a study of four aquatic plants that the root played a major role for the uptake of pollutants^55^. In a study of the phytoremediation of soil metals, Chaney *et al*. (1997) found Zn, Cd, and Ni concentrations are ten or more times higher in the root than the shoot^56^. Lu *et al*. (2004) showed that the highest concentration of cadmium (2044 mg/kg) and zinc (9652.1 mg/kg) was sequestered by the roots of water hyacinth compared to the shoot system (113.2 and 19626.7 mg/kg). They also found an increase in the relative growth rather than symptoms of stress in water hyacinth plants with treatments of 5 and 10 mg/L.

Arisz (1961) found that ions penetrated plants by passive process, mostly by exchange of cations which occurred in the cell wall^57^. Ionisation of various functional groups present on the surface of the adsorbents in aqueous solution facilitates cation binding with metal ions (Yao and Ramelow, 1997; Mahamdi and Nharingo, 2010 a,b; and Elangovan *et al*., 2008) in Sanmuga and Senmanthil^58^. Sharpe and Denny (1976) and Welsh (1961) showed however, that most metal uptake by plant tissue is by absorption to anionic sites in the cell walls and that the metals do not enter the living plant^59,60^.

Liao and Chang (2004) found that the Zn accumulation in the roots of water hyacinth was up to five times that of the shoot^61^. Over 97% of Cu absorbed by water hyacinth was located inside the roots. Translocation of Cd and Zn appeared to be much slower than sorption by roots, which could be a limiting factor for the bioconcentration of elements in shoots^30^. Heavy metals such as Cu and Zn are localised in cell walls, cell vacuoles and epidermal cell granules in association with anionic P and or S elements which eventually are complexed with phytochelatin, and their further encroachments into the interior cells of the bundle tissue are minimised or inhibited (Vesk *et al*.)^62^.

Work by Newete *et al*. (2016) demonstrated that over 80% of the total amount of metals removed was accumulated in the roots, of which 30–52% was adsorbed onto the root surfaces^31^. Furthermore, 73–98% of the total metal assimilation by water hyacinth was in the roots. Water hyacinth was found to be generally tolerant to metallotoxicity, except for Cu and Hg. It is thought that the high amount of removal of these elements is due to the formation of root plaques, which also contain in the main Fe. The formation of such plaques is a common feature of free-floating macrophytes^63,64^.

**Figure 9:**
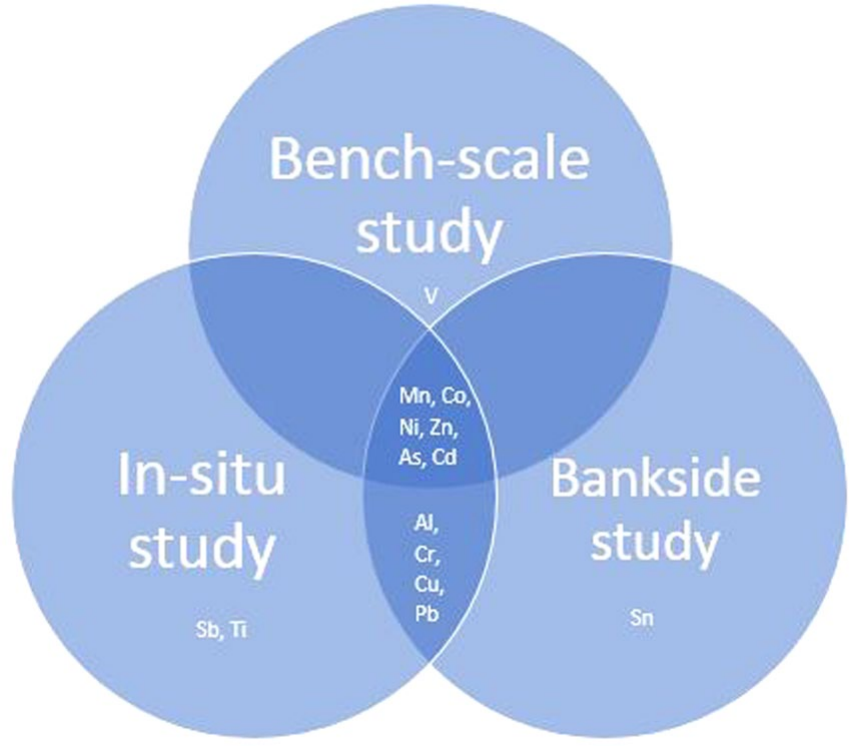
Venn diagram illustrating similarities and differences between metals removal during each of the three levels of study.

Figure 9 shows a Venn diagram illustrating the key differences and similarities in metals removal between each of the three studies. Whilst most metal removal takes place within the transition metals, i.e., those that have incompletely filled d orbitals and which readily form stable ions, removal is also evident for other metals, namely Al, Sn and Pb, which readily form ionic bonds with non-metals and finally, the metalloids As and Sb, which have similar electrical conductivities approaching those of metals^65–67^. In all three studies As, Cd, Co, Mn, Ni and Zn are removed by the water hyacinth. Additionally, Al, Cr, Cu and Pb are removed in both the *in-situ* and bankside studies. Then during each different level of study an additional metal, or metals are removed that were either not present, were below the limits of detection during the other two studies, or were present and not removed. Vanadium was removed during the bench-scale study, where Al, Ti and Cu were present though not removed and all other metals analysed for were either absent, or below the limit of detection. During the *in-situ* study, antimony and titanium are additionally removed in conjunction with As, Cd, Co, Mn, Ni, Zn, Al, Cr, Cu and Pb. All other metals were either absent, or below the limit of detection. Then, during the bankside study, tin is additionally removed in conjunction with As, Cd, Mn, Ni, Zn, Al, Cr, Cu and Pb and again, all other metals were either absent or below the limits of detection under the river conditions at that particular time. These key differences and similarities are further summarised within the supplementary data in Table S1.

It is also interesting to note that our findings of the uptake of metals are generally in agreement with the study by Shi and Xia (2010) who reported a suitable bioconcentration factor (BCF) of some of the elements, we investigated, in the roots of the water hyacinth plant^38^. For the elements we have investigated, that were also analysed by Shi and Xia, the order of BCF is as follows: Pb > Al > Co > Mn > Cd > As > Zn > Cr > Ni > Cu.

As with most types of remediation treatment, the question arises as to what do you do with the waste. Knowing whether metals are adsorbed onto, or assimilated within plant tissues and knowing their allocation between roots and shoots can inform how to re-treat biomass for metal recovery, or the mode of biomass reduction for safe disposal^31^. There are several options here, some of which are already being used in other parts of the world. Water hyacinth is found to have high nitrogen content and in combination with animal manure can be used for biogas production^68^. It has also reportedly been utilised in fertiliser manufacture, production of medicine, paper production, composting and in other bioenergy applications such as production of ethanol and in briquette form. The dried plant material is also widely used as a weaving material in manufacture of household baskets and furniture. Other researchers, for example Sinha *et al*. (2018) are trialling its use in removal of chromium and subsequent recovery of metals from the phytoremediation waste, once in sufficient concentration^69^.

There are of course, other aquatic plants that have known ability to remove metals, e.g. Water lettuce, Duckweed, Floating Pondweed etc. Previous work by other researchers already referenced within the present report^21,22,24,28,37^ has investigated the use of other plants in remediation, though the focus of this present study is in proving that an invasive weed that has desirable attributes, is free-floating and freely available in developing countries can be used as a sustainable phytoremediation technique.

The physical properties of water hyacinth, given its very long, fine, hairy root structure, with substantial associated surface area and prolific growth rate (doubling its biomass in just two weeks under optimum growth conditions) lends itself to the application of phytoremediation ideally. Yixiong and Xiumin (1990) have also theorised that along with the large surface area of the fibrous root, that microbes adhering to the roots may increase the level of metal accumulation^41^. The fact that the plant is regarded as an invasive weed presents a low-cost, or even free, natural source of material for remediation. Furthermore, the presently reported research demonstrates that it is still effective in regions beyond its normal habitat, though does not survive the winter months within the UK. These factors could potentially allow it to be used in conjunction with relevant permissions and biosecurity measures within a carefully controlled setting in the Northern Hemisphere, during the warmer months without fear of it becoming established and introduced into the wild.

There is a rich and diverse selection of literature available in the use and control of water hyacinth, including review articles and work of those such as Rezania, *et al*. (2015), Yan *et al*. (2016), Ncibi *et al*. (2017) and Kaur *et al*.^21,70–72^. However, there are several factors that make our study novel, this includes the following: Firstly, our study is the first to have investigated the potential of water hyacinth plant to clean a polluted river within the United Kingdom and indeed any country in northern Europe, or similar climatic zones in other parts of the world. Secondly, our study is the first paper to have reported phytoremediation using the water hyacinth plant at three different levels (bench, bankside and in-river). Thirdly, since the work of Shi and Xia (2010), this paper reports the potential of the water hyacinth plant to remove the second largest number of elements simultaneously present in river water (13 metallic/metalloid elements; from extensive review of the literature, the maximum number reported in previous studies was 18 by Shi and Xia (2010))^38^. To the best of our knowledge, this is the first study to have reported the potential of the water hyacinth plant to remove Sb, and only the second to have reported V removal from water since Shi and Xia (2010).

Vanadium pollution due to mining, poses a risk to human and animal health and our finding that the water hyacinth plant can remove V from water can be applied to address such pollution problems^73^. There is also increasing concern regarding antimony pollution and its toxic effects on ecosystems^74^. Our study, the first to show that the water hyacinth plant can be used to remove antimony from water, could be used to address antimony pollution of water in the future. We analysed a total of 21 elements, though some of the elements were either absent, or may have been present below the lower limits of detection (These are summarised within the supplementary information within Table S1). This represents the first report of the largest number of elements to be simultaneously investigated and removed in the presence of other nutrients. This has revealed that the water hyacinth plant can be highly versatile in removing elements from polluted water with diverse nutrient levels and the study provides valuable data for future research including the assessment of the mechanism of uptake.

Additionally, it should be noted that important differences between this work and that already reported within the literature are that these experiments have been conducted in the UK temperate climate toward the lowest end of the plant’s climatic tolerance, where the plant cannot survive naturally. To the best of our knowledge, an in-river test of this kind has never before been conducted within the Northern Hemisphere. The water chemistry of the Fendrod river is unique, given the dereliction that once blighted the landscape through which the river under investigation flows. The concentrations and oxidation state of the metals treated in this study will differ from those of previous studies, which have mainly examined synthetic single metal solutions in the relative absence of other nutrients. As well as synthetic solutions, the water treated here is actual polluted river water contaminated by a number of toxic metals present in excess of environmental quality standards.

### Strengths and limitations of study

Whilst recognising that it has its own set of limitations and will require further research and longer-term trials to fully assess and progress its full use, the presently presented research is the first report of a study that has expanded the phytoremediation use of a plant to a completely different climate. This study also provides a methodological advance since, in contrast to previous studies, it was conducted at the three different levels, namely at bench-scale, *in-situ* and at the river bankside via pump and treat. This type of research is timely and it is essential that the scientific community build upon these findings if we are to fully adapt to climate change and any associated environmental problems that this may bring, which may necessitate the sustainable management and use of such a prolific, yet ecologically admirable plant. This aspect of our work is very important and is made all the more relevant by related work undertaken by Kriticos and Brunel (2016), which looks at assessing and *managing* the emerging threat to the environment and water security presented by Water hyacinth and climate change^75^. Further development of our work, used in conjunction with some of the onward recovery and reuse options being developed, may offer just the type of radical solution required.

Even though the plant is highly invasive in certain climates, careful approaches can be developed to allow highly controlled use of the plant to remediate discretely polluted areas of rivers and waterbodies in Europe - to not use the amazing growth & phytoremediation potential of this plant, including its use in non-native environments such as the UK, will be a great loss to humanity. It is possible to exploit the properties of this invasive plant, which surely exists for some purpose, in a totally controlled manner, for example within a purpose-built treatment lagoon with inclusive biosecurity controls. However, development of this and similar techniques is beyond the scope of this proof of concept study and article.

Given the limited number of variables employed in these experiments, we recommend that longer-term studies be conducted, using differing controls to establish the effect of further variables such as plant growth rate and response to more extreme changes in water quality and climatic variation. The length and timing of these studies was largely dictated by current UK legislation, though an exemption and relevant permits could be obtained for longer term trials. Differing controls should also be utilised in the form of other plant types, both native and non-native, as well as further detailed analysis of the metal content and other compositional characteristics of the plants in order to draw effective comparison.

## Conclusions

We have demonstrated for the first time that it is possible to extend the phytoremediation potential of the fastest growing aquatic plant in the world within a completely different climatic region. This has important connotations from a climate change perspective in relation to future plant distribution. We have demonstrated the potential of *E. crassipes* for removing a range of toxic metals from polluted water in the below-optimal, temperate, maritime UK climate. The results of all three studies are very encouraging and lays the foundation for future research. The bench-scale study demonstrated excellent metals removal potential, in some cases of up to 100% over three weeks. Furthermore, the *in-situ* and bankside experiments confirm this potential in a dynamic environmental setting. It is recommended that the principles of both the *in-situ* and bankside study be further refined and subsequent trials be repeated in more favourable conditions, perhaps within a purpose-built treatment lagoon.

The impact of this seminal piece of research has potentially far-reaching consequences in that it offers the possibility of using an invasive weed, which is a widespread global problem, to provide a low-cost, low energy remediation technology, that has the added benefit of being freely available in developing countries and that is harvested in attempts to control its proliferation. The plant, although invasive has been shown to be effective in a lower temperature climate, though will not survive the winter months, which in turn acts as a control which may allow its use in countries where it is not currently permitted to be introduced. This compared to the footprint and cost of chemical and other remediation technologies surely merits its full exploitation.

## Electronic supplementary material

Supplementary Information
