# Supplementary material for: Extending the geographic reach of the water hyacinth plant in removal of heavy metals from a temperate Northern Hemisphere river
Source: Sci Rep. 2018 Jul 23;8:11071. doi: 10.1038/s41598-018-29387-6 (PMC6056511; doi:10.1038/s41598-018-29387-6)
Supplement: Supplementary file 1 — Supplementary Information [file 41598_2018_29387_MOESM1_ESM.pdf]

Supplementary Information in support of the research article "Extending the geographic reach of the water hyacinth plant in removal of heavy metals from a temperate Northern Hemisphere river"

Jonathan L Jones[1, 2], R O Jenkins[1], and Parvez I Haris[1]*
[1]Faculty of Health & Life Sciences, De Montfort University, The Gateway, Leicester, LE1 9BH, United Kingdom
[2]Natural Resources Wales, Maes Newydd, Britannic Way West, Llandarcy, Neath-Port Talbot, SA10 6JQ
*Corresponding author: Professor Parvez Iqbal Haris. E-mail: pharis@dmu.ac.uk

Element analysed using ICP-MS	Bench-scale study (Figures denote Mean percentage removal 3 weeks exposure)			In-Situ study (Figures denote Mean percentage removal 8 hours exposure)			Bankside study (Figures denote Mean percentage removal 4 hours exposure)		
	Present (removed)	Present (not removed)	Absent/ below LOD*	Present (removed)	Present (not removed)	Absent/ below LOD	Present (removed)	Present (not removed)	Absent/ below LOD
Be			X			X			X
Al		X		X (18.23%)			X (24.58%)		
Ti		X		X (10.41%)					X
V	X (100%)					X			X
Cr			X	X (39.56%)			X (22.57%)		
Mn	X (77.36%)			X (6.37%)			X (5.38%)		
Co	X (100%)			X (21.39%)			X (6.88%)		
Ni	X (41.09%)			X (7.18%)			X (5.76%)		
Cu		X		X (12.81%)			X (10.76%)		
Zn	X (81.31%)			X (12.22%)			X (4.04%)		
As	X (43.88%)			X (6.08%)			X (9.39%)		
Se			X			X			X
Mo			X			X			X
Ag			X			X			X
Cd	X (97.94%)			X (15.47%)			X (6.72%)		
Sn			X			X	X (8.32%)		
Sb			X	X (26.49%)					X
Te			X			X			X
Tl			X			X			X
Pb			X	X (7.22%)			X (25.54%)		
U			X			X			X

Supplementary Table S1 – Showing differences and similarities between each of the three studies conducted.

Legend - *LOD = Limit of Detection

	Common to all three studies
	Common to two of the studies
	Unique to a particular study

Mean Percentage removal figures shown for information only and should not be directly compared across the 3 studies due to differing variables and exposure times.

Bench-scale study In-situ study Bankside study

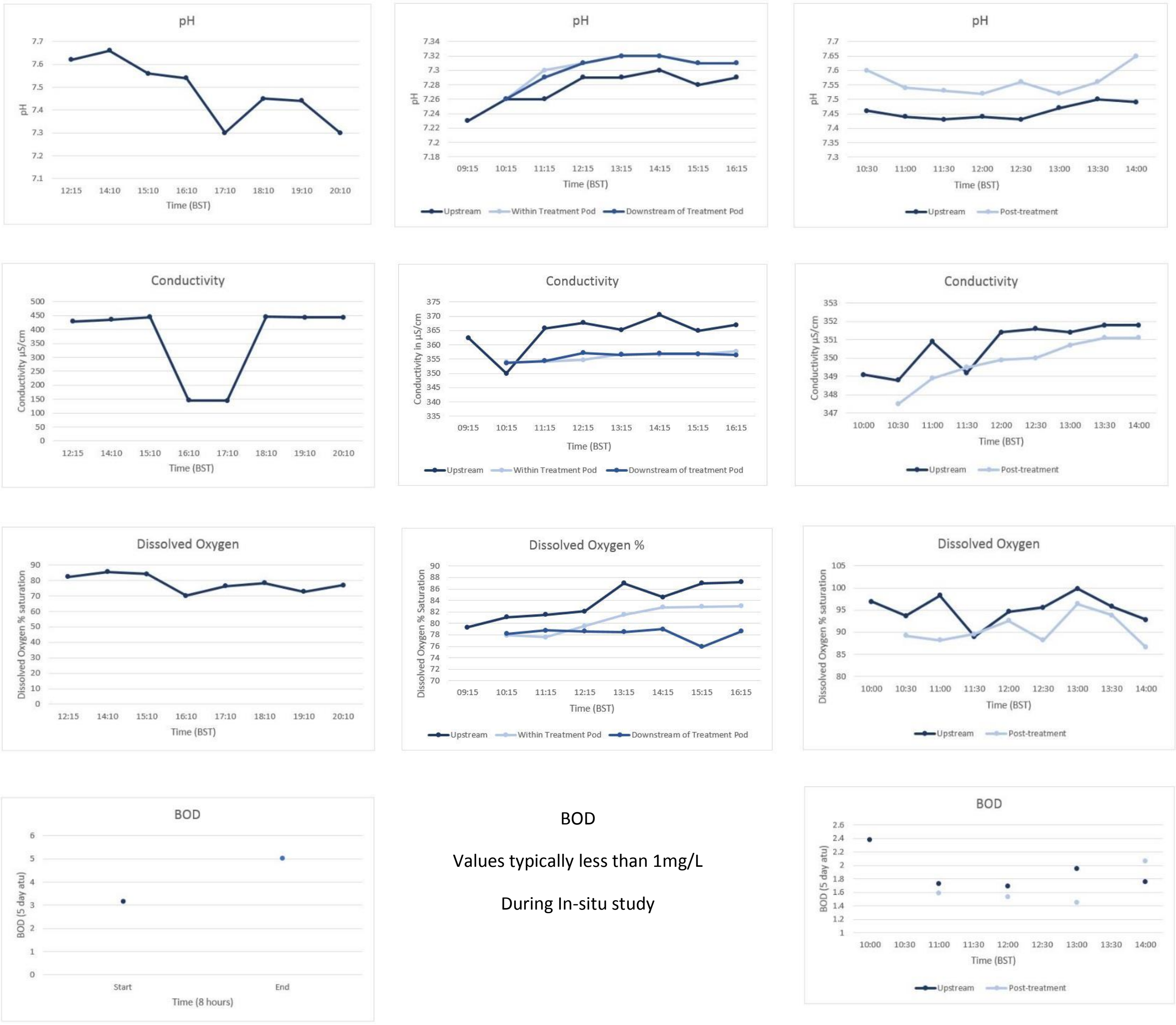

Supplementary Figure S1 – A selection of time series data from each of the three studies illustrating some of the changes observed in water chemistry (pH, Conductivity, Dissolved Oxygen and Biological Oxygen Demand.

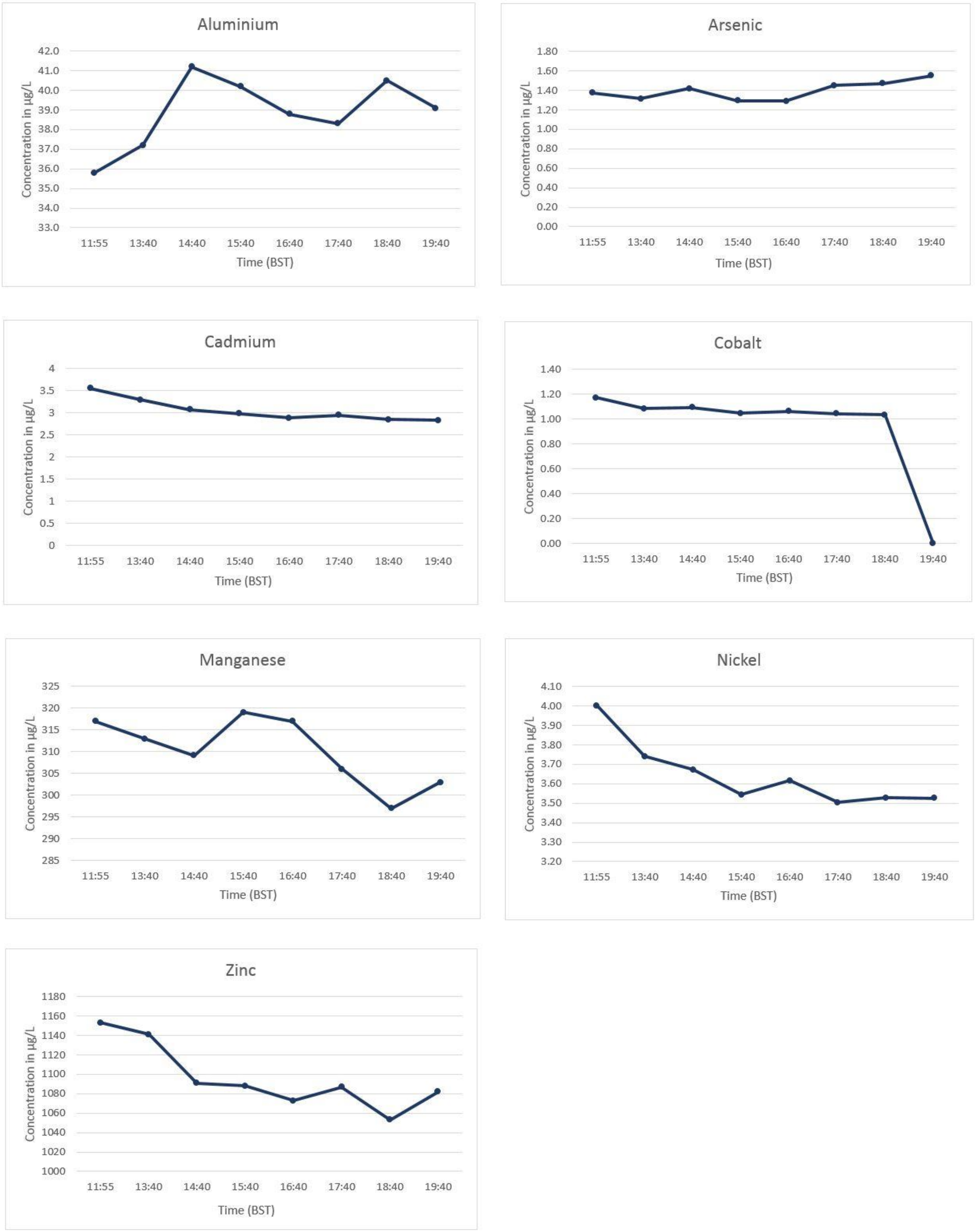

Supplementary Figure S2 – A selection of time series data showing metals removal observed during the bench-scale study.

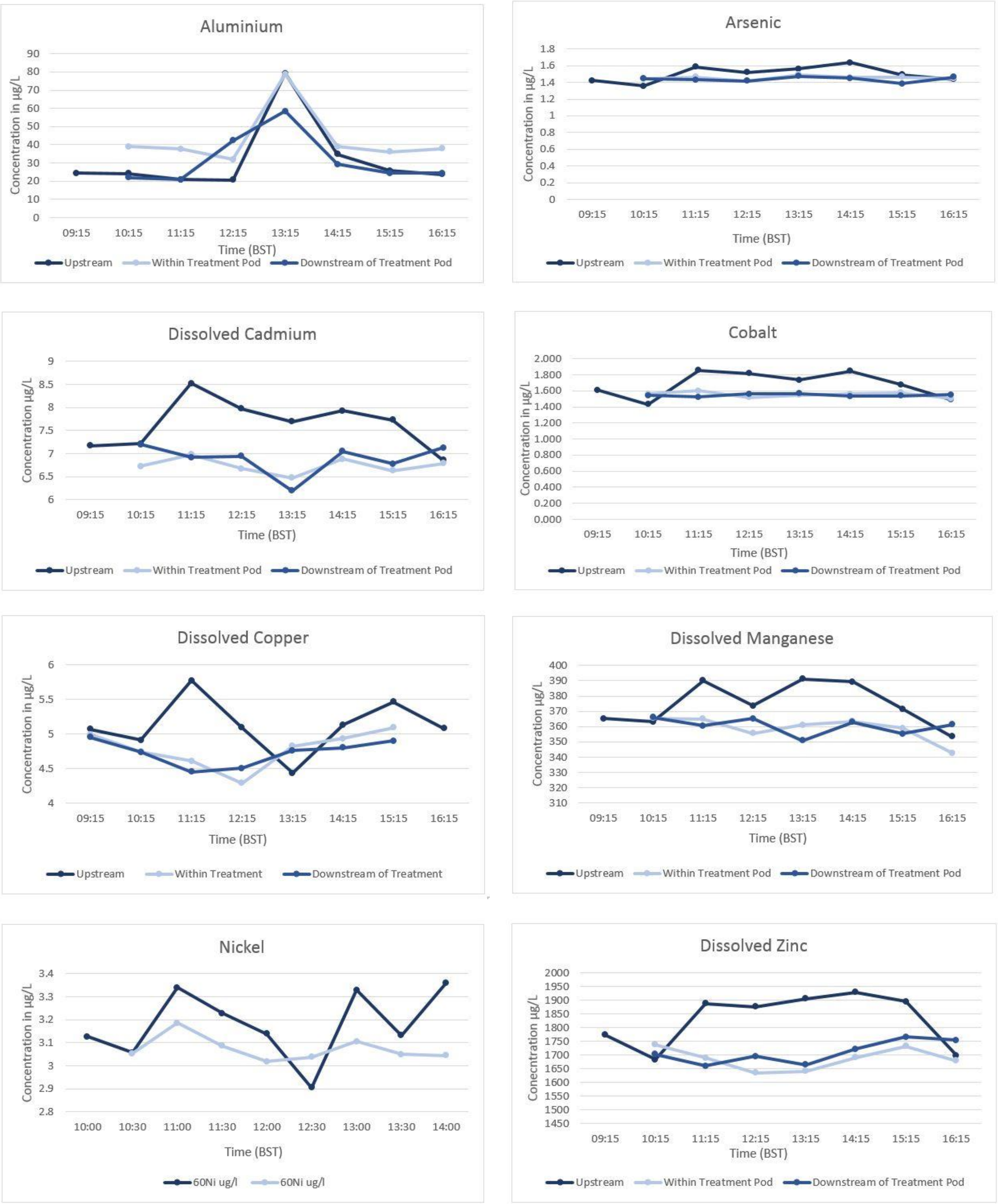

Supplementary Figure S3 – A selection of time series data showing metals removal observed during the in-situ study.

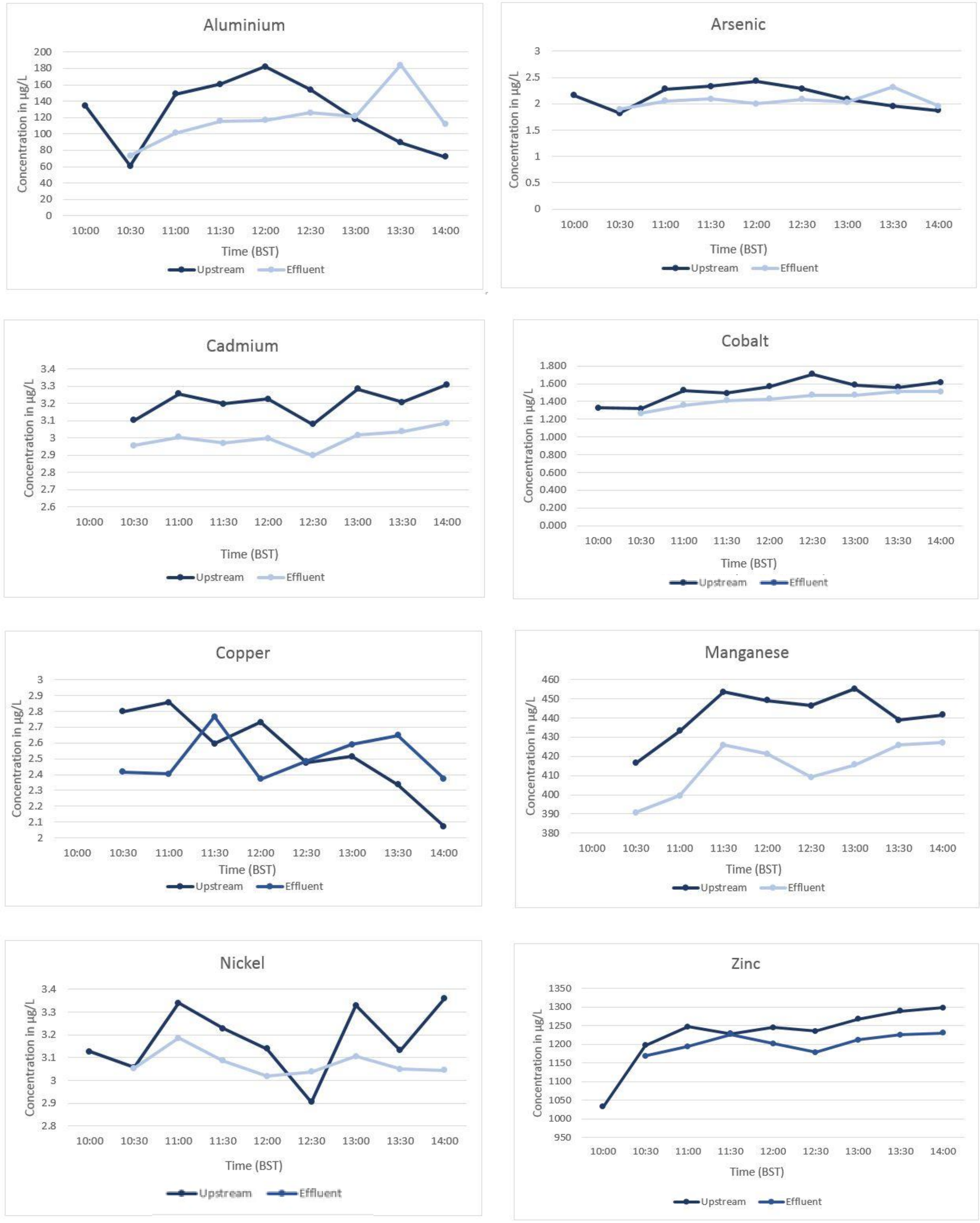

Supplementary Figure S4 – A selection of time series data for metals removal observed during the Bankside study.
